# Amaranth oil application for coronary heart disease and hypertension

**DOI:** 10.1186/1476-511X-6-1

**Published:** 2007-01-05

**Authors:** Danik M Martirosyan, Lidia A Miroshnichenko, Svetlana N Kulakova, Ala V Pogojeva, Vladimir I Zoloedov

**Affiliations:** 1 Functional Foods Center, Dallas, TX, USA; 2 Voronezh State University, Voronezh, Russia; 3 State Institute of Nutrition of the Russian Academy of Medical Sciences, Moscow, Russia

## Abstract

Cardiovascular disease (CVD) is the Nation's leading killer for both men and women among all racial and ethnic groups. Development and progression of CVD is linked to the presence of risk factors such as hyperlipidemia, hypertension, obesity, and diabetes mellitus. It is known that cholesterol is an indicator of increased risk of heart attack and stroke. Low-density cholesterol (LDL) above 130 mg/dl high-density cholesterol (HDL) cholesterol below 35 mg/dl and total blood cholesterol above 200 mg/dl are indicators of problematic cholesterol. Proper ranges of cholesterol are important in the prevention of CVD.

It has been suggested that a reduction in the consumption of saturated and an increase in unsaturated fatty acids is beneficial and prevents CVD. Amaranth grain contains tocotrienols and squalene compounds, which are known to affect cholesterol biosynthesis. The cholesterol precursors squalene, lanosterol and other methyl sterols, reflect cholesterol synthesis [1-3], whereas plant sterols and cholestanol, a metabolite of cholesterol, reflect the efficiency of cholesterol absorption in normal and hyperlipidemic populations [4-6].

Qureshi with co-authors [7] showed that feeding of chickens with amaranth oil decreases blood cholesterol levels, which are supported by the work of others [8]. Previously, we have shown that Amaranth oil modulates the cell membrane fluidity [9] and stabilized membranes that could be one reason as to why it is beneficial to those who consume it. It is known that in hypertension, the cell membrane is defective and hence, the movement of the Na and K ions across the cell membranes could defective that could contribute to the development of increase in blood pressure. Based on these properties of amaranth oil we hypothesize that it could be of significant benefit for patients with CVD.

## Background

Amaranth is defined as a "never-fading flower" in Greek. Various **Amaranthus species were grown by the Aztecs 5,000 to 6,000 years ago, prior to the disruption of the South American civilization by the Spanish conquistadors**. Both the grain amaranth and leaves are utilized for use for human as well as for animal food [10]. The nutritional value of amaranth has been extensively studied [11-14]. Grain amaranth has higher protein than other cereal grains and has significantly higher lysine content [15,16]. It has been shown that amaranth leaves are an excellent source of protein, with its maximal accumulation in the blossoming phase [17] (17.2–32.6% from dry weight for varius samples).

Amaranth grain consists of 6 to 9% of oil which is higher than most other cereals. Amaranth oil contains approximately 77% unsaturated fatty acids and is high in linoleic acid, which is necessary for human nutrition. The lipid fraction is unique due to the high squalene content. Detailed studies on amaranth grain oil have been researched further in the last 2–3 decades [18-21]. Vegetable amaranth has received significantly less research attention than grain amaranth. However, it has been rated considerably higher in minerals, such as calcium, iron, phosphorous [22,23] and caretonoids [24] than most vegetables. Pharmacological properties of different amaranth species also have been investigated. It was determined that Amaranth paniculatus and Amaranth cruentus are good sources of flavanoids, especially for rutin, which are mostly produced in the stage of blossoming [25]. Usage of amaranth as livestock feed indicated relatively high protein qualities [26-28].

The vital issue with the future of amaranth can be determined by the end-use product. Since 1991, we are working on vegetable and grain amaranth commercialization by formulating and producing new amaranth products especially for the prevention and treatment of cardiovascular diseases such as coronary heart disease and hypertension. Our investigation indicated that amaranth grain is a good source of modern diet, particularly to make special products for the people who are at a high risk to cardiovascular diseases [29]. Furthermore, new formulas were created on the base of natural ingredients only with amaranth flour, containing a significant amount of magnesium and dietary fiber. This is extremely beneficial for people who have high blood pressure and are at a high risk of cardiovascular diseases such as heart attack [30].

Coronary heart disease is the most common cause of mortality. Risk factors for CVD include: hyperlipidemia, hypertension, obesity, and glucose intolerance. Several studies revealed that a reduction in blood pressure reduces the risk and incidence of CVD [31,32].

Studies clearly established that decreased consumption of saturated and trans fats is of significant benefit to those who have hypertension. In this context, it important to mention that consumption of products with low content of animal fat and increased use of unsaturated fats are of benefit in hypertension and may reduce the risk of CVD. Amaranth oil can lower cholesterol in animal models in view of its rich content of squalene and polyunsaturated fatty acids. This is supported by the observation that hamsters which received hypercholesterolemic diets and were given Amaranth oil (5%), they experienced a decrease in total and non-high-density lipoprotein cholesterol by 15 and 22%, respectively, in comparison to the control group [33]. In the presented study, we investigated the effects of amaranth oil in patients suffering from coronary heart disease (CHD) and hypertension (HP) with obesity.

## Methods and materials

The effect of amaranth oil was studied in a randomized placebo-controlled clinical trial of 125 patients with CVD. The patients were randomized to amaranth oil (3 ml amaranth oil provides a 100 mg squalene) 3–18 ml amaranth oil daily. All 125 patients got similar dietary and behavioral recommendations and low-salt diet. Biochemical and clinical indices was monitored during the treatment and after 3 weeks. The experiment was conducted on men and women, aged 32–68, suffering from coronary heart disease and hypertension accompanied by obesity. The following exclusion criteria were used:

1.	Those having been affected with stroke, myocardial infarction, coronary angioplasty or having undergone an operation of aortocoronary shunting three months prior to the investigation.

2.	Diagnosis of acute coronary deficiency three months prior to the investigation.

3.	Renal insufficiency or liver failure.

4.	The presence of acute or aggravated chronic diseases.

Eighty patients (85 people in the main group and 40 people in the group of comparison) suffering from coronary heart disease and hypertension in the 1^st ^and 2^nd ^stages accompanied by obesity of the 1^st ^and 3^rd ^stages were under observation.

All the patients received a reduced hyposodium antiatherogenic diet (HAD). The test group of patients received 3, 6, 12 and 18 ml. of amaranth oil instead of the sunflower oil. The control group received only the HAD.

Amaranth oil has been extracted from the Amaranthus hybridus L. (Amaranthaceae). Amaranth oil was applied in four doses by 3 ml. (100 mg squalene – 25% adequate level of consumption), 6 ml. (200 mg), 12 ml. (400 mg. – 100%) and 18 ml. (600 mg. – 150%) per day.

The overall duration of the clinical trial was 3 weeks. All the patients received a traditional course of treatment, which included the hyposodium antiatherogenic diet, exercise, and hydro and procedures. The test group and the control group were made of patients suffering from coronary heart disease and hypertension of the 1^st ^and 2^nd ^stages. A thorough medical examination was conducted. An electrocardiogram (ECG) and echocardiogram were conducted for the verification of the diagnosis. All patients in the test group were closely followed for any possible side effects due to the consumption of amaranth oil. Their organoleptic properties and tolerance were evaluated based on the availability and intensity of side effects as a result of the intake of the product. The clinical trials were conducted on the following groups of patients:

The patients of the comparison group – 40 people (2 male and 38 females) suffering from CHD and HP (average age was 52.4 years old). The patients of the first main group – 25 people (5 males and 20 females) suffering from CHD and HP (average age was 59.4 years old). They got a ration where 20 g sunflower oil have been replaced by amaranth oil and corn oil. The amount of squalene in this mix was 100 mg per a day. The patients of the second main group (200 mg squalene/day) – 20 patients (2 males and 18 females) suffering from CHD and HP (average age was 46.6 years old). The patients of the third main group (400 mg squalene/day) – 20 patients (3 males and 17 females) suffering from CHD and HP (average age was 59.2 years old). The patients of the forth main group (600 mg squalene/day) – 20 patients (3 males and 17 females) suffering from CHD and HP (average age was 59.2 years old).

### Description of Hyposodium Antiatherogenic Diet

The hyposodium antiatherogenic diet (HAD) is characterized by its low content of calories, small amounts of salt and fat, refined carbohydrates, cholesterol-containing products and extracted substances. The protein content in the ration corresponds to the physiological standard. It includes products containing lipotropic substances, polyunsaturated fatty acids, and food fibers. The description of the HAD diet is described below in Table 1.

**Table 1:** Chemical Composition of HAD and Rations including Amaranth Oil

**Indices**	**HAD**	**Amaranth Oil **(3 g/day)	**Amaranth Oil **(6 g/day)	**Amaranth Oil **(12 g/day)	**Amaranth Oil **(18 g/day)
Proteins, g	75	75	75	75	75
Fats, g	60	60	60	60	60
Carbohydrates, g	190	190	190	190	190
Caloricity, kcal	1600	1600	1600	1600	1600
Fatty acids:					
Unsaturated fatty acids	22.831	23.217	23.502	24.173	24.844
C 14:0	5.054	5.062	5.065	5.076	5.087
C 15:0	0.164	0.164	0.172	0.18	0.188
C 16:0	11.844	12.646	12.523	13.202	13.881
C 17:0	0.288	0.318	0.35	0.412	0.474
C 18:0	5.062	4.682	5.007	4.952	4.897
C 20:0	0.231	0.297	0.222	0.213	0.204
C 22:0	0.188	0.048	0.163	0.138	0.113
Monounsaturated fatty acids, g	21.423	22.515	21.285	21.147	21.009
C 14:1	0.455	0.455	0.455	0.455	0.455
C 16:1	1.852	1.879	1.86	1.868	1.876
C 17:1	0.008	0.008	0.008	0.008	0.008
C 18:1	18.707	19.708	18.515	18.323	18.131
C 20:1	0.207	0.241	0.253	0.299	0.345
C 22:1	0.194	0.224	0.194	0.194	0.194
Polyunsaturated fatty acids	15.129	13.724	14.32	13.511	12.702
C 18:2	13.424	11.761	12.560	11.696	10.832
C 18:3	0.275	0.533	0.330	0.385	0.440
C 18:4	0.015	0.015	0.015	0.015	0.015
C 20:4	0.355	0.355	0.355	0.355	0.355
C 20:5	0.042	0.042	0.042	0.042	0.042
C 22:5	0.902	0.902	0.902	0.902	0.902
C 22:6	0.116	0.116	0.116	0.116	0.116
PUFAs ω-6/PUFAs ω-3	10.2	7.533	9.192	8.254	7.384
∑ PUFAs ω-3	1.575	1.608	1.405	1.460	1.515

### Clinical methods of investigation

An integrated checkup of the patients in the Clinic included the study of signs for diseases such as: blood arterial pressure (BAP) level, heartbeat rate (HBR), EKG data, and the evaluation of organoleptic properties and tolerance to oil.

### Biochemical methods of investigation

The biochemical indices were determined by means of the "SPECTRUM" analyzer (ABBOTT, USA). The content of low-density cholesterol in the blood serum, very low-density cholesterol (VLDL), and atherogenic coefficient value (AC) were evaluated in the rated manner.

VLDL = TG/2.2

LDL = Total Cholesterol - TG/2.2 - HDL

AC = (Total Cholesterol - HDL)/HDL

The statistical treatment of the results was recorded in EXCEL, and was expressed as M ± m. The significant level of the revealed differences was determined by using Student's t-criteria.

## Results and discussion

The amaranth oil was extracted by press methods and the following physical and chemical characteristics were determined. The results are shown in Table 2.

**Table 2:** Physico-Chemical Characteristics Of Amaranth Oil

Physico-Chemical Characteristics	**Characteristics of Amaranth Oil**
Iodine number, mg	130
Acid number, mg KOH/g,	2.9
Mass fraction of the non fat admixtures in %	0.2
Mass fraction of the phosphor containing substances in P_2_O_5_%	0.1
Mass fraction of moisture and volatile substances, %	0.1
Peroxide number, mmole/kg of active oxygen	2.4

It is known that amaranth grain contains 6 to 10% oil, which is mostly within the germ [34-36]. Amaranth oil is predominantly an unsaturated oil (76%) and is high in linoleic acid, which is necessary for human nutrition. Yanez E. with coauthors [37] showed that oil extracted from Amaranthus cruentus contained 19% palmitic acid, 3.4% stearic acid, 34% oleic acid and 33% linoleic acid. Docosaenoic acid (C22: 1) was present at the level of 9%. The ratio of saturated to unsaturated fatty acids was approximately 1:3. We provided an extraction of amaranth oil by press methods for the experiments. The fatty acid composition of amaranth oil was determined, which is provided in Table 3.

**Table 3:** Fatty Acid Composition of Amaranth Oil

**Fatty acids (Shorthand designation)**	**Mass Fraction of Fatty Acids (in %)**	
	
	Press methods	Chemical extraction
14:0	0.14	0.15
15:0	0.15	0.08
16:0	19.19	18.59
16:1	0.13	0.08
17:0	1.05	1.37
18:0	3.38	4.45
18:1	22.64	22.69
18:2	49.89	48.00
18:3ω6	0.35	0.35
18:3ω3	1.01	0.92
20:0	0.16	0.27
20:1	1.04	1.49
22:0	0.32	0.24
24:0	0.05	0.08
22:1	0.07	0.7
24:1	0.43	0.54

The investigation of He HP and others has shown that the major fatty acids in Amaranth oil consist of palmitic acid (19.1–23.4%), oleic acid (18.7–38.9%), and linoleic acid (36.7–55.9%) [38]. The food and energy values of amaranth oil are shown in Table 4. As you can see, amaranth oil received by the press method has a great amount of squalene and tocopherols. It is known that squalene is an intermediate metabolite in the synthesis of cholesterol. Supplementation of squalene to mice has resulted in marked increases in cellular and non-specific immune functions in a dose-dependent manner [39]. Experimental evidence suggests that 90 percent of the newly formed squalene is stored in the lipid droplet and only 10 percent is used in cholesterol synthesis [40].

**Table 4:** Food and Energy Value of 100 g Amaranth Oil

**Food value**	**Amaranth Oil**
Triglycerides (g)	78
Squalene (g)	5.9
Phospholipids (g)	8
Phytosterols (g)	2
Sum of tocopherols (vitamin E), in mg	300
Carotinoids (mg)	0.5
Energy value, kcal/kJ	711/87

### Evaluation of organoleptic properties and tolerance of amaranth oil

Based on a standard procedure, an extended testing of amaranth oil was made. The evaluation of its organoleptic properties and tolerance was conducted based on a fill-in-the-blank method. The organoleptic properties were evaluated based on 5 parameters of a five-score system. In addition, the tolerance of the oil was tested. The results received are presented in Tables 5 and 6 below.

**Table 5:** Value Of Amaranth Oil's Organoleptic Properties

**Indices**	**Number of Scores**	**Amaranth Oil**
Outward Appearance	5 4	100% 0
Odor	5 4	83% 17%
Color	5 4	98% 0
Taste	5 4	92% 8%
Consistence	5 4	100% 0

**Table 6:** Evaluation Of Tolerance Of Amaranth Oil

**Indices**	**Amaranth Oil **3 g (100 mg squalene per day)	**Amaranth Oil **6 g (200 mg squalene per day)	**Amaranth Oil **12 g (400 mg squalene per day)	**Amaranth Oil **18 g (600 mg squalene per day)
Eructation	0	0	0	0
Nausea	0	0	0	0
Heartburn	0	0	0	0
Bitter taste in mouth	0	0	0	0
Pains in the abdomen	0	0	0	0
Allergic reactions	0	0	0	0

As can be seen in Table 5, the overwhelming majority of patients (over 80%) highly enjoyed (put the highest score for) the oil quality. Much emphasis was made on its beneficial organoleptic properties. Pleasant outward appearances, homogeneous consistence and the absence of foreign inclusions were also established. The properties of the amaranth oil met the required standards. A growing number of the patients placed a lot of emphasis on its amiable taste.

The percents indicate the number of people polled. None of the participants taking part in the testing mentioned an unpleasant "aftertaste". As can be seen in Table 6, the tolerance of amaranth oil was good. Throughout the clinical trials, no single case of intolerance, dyspepsia occurrences, allergic reactions or other side effects were established. Upon the completion of the clinical trials, all of the patients expressed their willingness to continue the intake of the certified amaranth oil under house conditions.

It has been reported that a combination of fish oil supplementation and salt restriction is highly effective in lowering systolic and diastolic blood pressure [41]. After four weeks the mean systolic blood pressure had decreased by 8.9 mm Hg and the diastolic pressure by 6.0 mm Hg.

The researchers concluded that sodium restriction combined with fish oil supplementation lowered blood pressure in elderly people with normal blood pressure. It has been shown that supplementation with 7.7 to 9 grams/day of fish oil will reduce systolic blood pressure by 4 mm Hg and diastolic pressure by 3 mm Hg in hypertensive individuals [42]. Table 7 shows how the systolic blood pressure decreased with the amaranth oil treatment combined with the HAD.

**Table 7:** Dynamics of Clinical Characteristics under the Effects of Diet and Amaranth Oil

**Clinical Attributes**	**HAD**		**Amaranth Oil **3 g (100 mg squalene per day)	**Amaranth Oil **6 g (200 mg squalene per day)	**Amaranth Oil **12 g (400 mg squalene per day)	**Amaranth Oil **18 g (600 mg squalene per day)
Systolic Blood Pressure (mm Hg)	1	146 ± 2.13	143 ± 7.58	142 ± 2.48	149 ± 2.42	144 ± 1.13
	2	121 ± 2.08**	118 ± 2.28**	116 ± 2.92**	120.6 ± 1.40**	114 ± 1.12**
Diastolic Blood Pressure (mm Hg)*	1	96.8 ± 2.59	87.5 ± 3.61	98.1 ± 1.01	97.5 ± 2.13	89.0 ± 2.01
	2	82 ± 1.8*	76.7 ± 1.36*	85.2 ± 1.86**	80.9 ± 0.8**	72.0 ± 2.11**
Frequency of Heart Contraction (hit/min)	1	82.2 ± 2.50	76.2 ± 2.8	78.2 ± 2.23	78.8 ± 2.13	79.4 ± 2.15
	2	69.3 ± 2.92	68 ± 2.7	67.4 ± 2.94	68.0 ± 2.17	78.3 ± 2.02
Weight (kg)	1	93.4 ± 2.51	85.9 ± 4.138	97.9 ± 3.98	117.9 ± 4.66	91.3 ± 3.14
	2	87.7 ± 1.98	81.2 ± 3.28	91.5 ± 3.82	110.6 ± 3.40	86.7 ± 1.03

1 – Before Treatment, 2 – After Treatment

The weight loss for the control group and the group of patients with an amaranth diet did not have a big difference. The weight loss was about 5.1–6.5%, which is related to the HAD.

### Evaluation of the dynamics of the clinical indices during diet therapy with the inclusion of amaranth oil

The diet therapy with amaranth oil contributed to the decrease or disappearance of headaches, weakness, increased fatigability, shortness of breath during a physical activity, edema of legs toward the evening hours and feeling of intermission of heart function in most patients.

The positive dynamics on electrocardiograms were observed in 40–50% of patients and were displayed by rhythm normalization (disappearance of sinus tachycardia, bradycardia, single ventricular and supraventricular extrasystole), decrease in the intensity of the signs of coronary deficiency (which was evident by the change in the interval S-T and wave T).

Results given in Table 7 shows that the level of systolic arterial pressure was decreased by 18%, 19%, and 21% in the course of the treatment in patients of the 1^st ^, 2^nd ^and 3^rd ^of the main group and the groups of comparison – by 18%, diastolic – by 14%, 15%, 19% and by 17%. In addition, heart rate in the course of the treatment tended to decrease in patients of all the groups.

The degree of decrease in excess body weight in the course of the treatment of the patients in the main groups and the groups of comparison amounted to 6.5%, 6.2%, 5.1% and 6.1%. The average daily loss of body weight for the patients of the main groups and the groups of comparison was equal to 304 g, 348 g, 219 g and 271 g, accordingly.

### Evaluation of the dynamics of biochemical indices during diet therapy with the inclusion of amaranth oil

It is known that there is a correlation between diet and cholesterol level in blood serum. Alpha-linolenic acid (ALA), omega-3 fatty acid, found in large amounts in flaxseed oil is not effective in lowering triglyceride levels, a risk factor for heart disease [43]. A group of researchers conducted a study in people with a history of heart disease, using the Mediterranean diet [44]. The Mediterranean diet included a special margarine high in alpha-linolenic acid. Those people assigned to the Mediterranean diet had a 70% reduced risk of dying from cardiovascular diseases compared with the control group during the 27 months. Similar results were reported by others as well [45]. Nevertheless, the success of the Mediterranean diet does not prove that ALA protects against heart disease [46]. The influence of an antisclerotic diet with a sour-milk product enriched with an extract of leaves of an amaranth in patients with ischemic heart disease and hypertension was investigated by Zoblkova ZS and coauthors [47]. As a result of the diet with extracts of amaranth leafs were positive dynamic of clinic manifestation and lipid spectrum of blood.

It is evident from Table 8 that inclusion of amaranth oil in the HAD contributed to a statistically significant decrease in the total cholesterol level in the blood serum in patients of the 1^st ^, 2^nd ^and 3^rd ^of the main groups and the groups of comparison, accordingly, by 14%, 17%, 20% and 12% and triglycerides and VLDL Cholesterol – by 13%, 21%, 36% and 16%, LDL – by 19%, 23%, 25% and 12%, the value of atherogenic ratio – by 18%, 23%, 32% and 8%. The contents in the blood serum HDL in patients of the 3^rd ^main group had a tendency towards increase, in the main groups it virtually remained unchanged, and in the group of comparison it decreased by 9%. The level of the remaining investigated biochemical indices changed to the same degree in the patients of the main groups and the group of comparison. It is known, that low HDL cholesterol and high LDL cholesterol are more specifically linked to cardiovascular disease than is total cholesterol [48].

**Table 8:** Dynamics of biochemical characteristics of patients under the effects of diet and amaranth oil

**Biochemical Sings**	**HAD**		**Amaranth Oil **3 g (100 mg squalene per day)	**Amaranth Oil **6 g (200 mg squalene per day)	**Amaranth Oil **12 g (400 mg squalene per day)	**Amaranth Oil **18 g (600 mg squalene per day)
Total cholesterol mmol/L	1	6.31 ± 0.23	7.06 ± 0.6	5.58 ± 0.23	5.76 ± 0.23	6.60 ± 0.23
	2	5.57 ± 0.20*	5.69 ± 0.3*	4.79 ± 0.20*	4.76 ± 0.20*	5.29 ± 0.20*
Triglycerides mmol/L	1	1.34 ± 0.13	1.57 ± 0.2	1.88 ± 0.13	1.17 ± 0.13	2.49 ± 0.13
	2	1.13 ± 0.10	1.45 ± 0.18	1.62 ± 0.10	0.93 ± 0.10	1.58 ± 0.10
HDL Cholesterol mmol/L	1	1.65 ± 0.11	1.66 ± 0.32	1.16 ± 0.11	1.26 ± 0.11	1.15 ± 0.11
	2	1.51 ± 0.07	1.42 ± 0.22	1.13 ± 0.07	1.24 ± 0.07	1.19 ± 0.07
LDL Cholesterol mmol/L	1	4.03 ± 0.20	4.69 ± 0.44	3.90 ± 0.20	3.74 ± 0.20	4.42 ± 0.20
	2	3.54 ± 0.17	3.61 ± 0.26	3.18 ± 0.17	2.89 ± 0.17	3.32 ± 0.17
VLDL Cholesterol mmol/L	1	0.61 ± 0.06	0.71 ± 0.09	0.99 ± 0.06	0.58 ± 0.06	1.25 ± 0.06
	2	0.51 ± 0.04	0.66 ± 0.08	0.81 ± 0.04	0.46 ± 0.04	0.79 ± 0.04
Aterogenic Index (AI)	1	3.15 ± 0.27	3.65 ± 0.41	4.06 ± 0.27	3.80 ± 0.27	5.21 ± 0.27
	2	2.90 ± 0.25	3.47 ± 0.44	3.33 ± 0.25	2.98 ± 0.25	3.54 ± 0.25

1 – Before Treatment, 2 – After Treatment

AI = (Total Cholesterol - HDL): HDL under p < 0.05; ** under p < 0.01.

The link between high triglyceride levels and cardiovascular diseases is not as well established as the link between high cholesterol and heart disease. But, according to some studies, a high triglyceride level is an independent risk factor for cardiovascular diseases in some people [49].

It is known that squalene is involved in cholesterol synthesis, but it is not clear yet squalene's role in the process of lowering the cholesterol amount in blood serum. Even more, dietary supplementation with one gram of squalene daily for nine weeks was reported to cause increases in VLDL and LDL-cholesterol concentrations by 34 and 12 percent, respectively. However, a subsequent six-week period on a lower dose of squalene dietary supplementation (0.5 g/day) normalized serum sterols [50].

### Evaluation of fatty-acid composition of cell membrane in patients with coronary heart disease and hypertension

Studies have shown that replacing saturated fat with unsaturated fat in the diet can help lower blood pressure. For example, Fish oils containing eicosapentaenoic acid (EPA) and docosahexaenoic acid (DHA) have been found more effective in lowering triglyceride levels and blood pressure [51].

The analysis of the fatty acid composition of erythrocyte membrane of patients was conducted based on a traditional method of gas-liquid chromatography. The investigation results are presented in Table 9 below.

**Table 9:** The effect of amaranth oil on fatty-acid composition of erythrocyte membrane of patients

**Fatty Acids**		**HAD**	**Amaranth Oil **3 g (100 mg squalene per day)	**Amaranth Oil **6 g (200 mg squalene per day)	**Amaranth Oil **12 g (400 mg squalene per day)	**Amaranth Oil **18 g (600 mg squalene per day)
12:0	1	--	0.35	1.14	1.22	1.07
	2	--	0.34	0.57	1.26	0.90
14:0	1	2.02	5.2	2.18	2.23	2.19
	2	1.44	4.11	0.97	2.89	1.99
15:0	1	2.9	2.28	0.61	0.62	0.77
	2	2.08	2.29	0.44	0.76	0.72
16:0	1	15.9	24.99	15.4	18.44	19.6
	2	17.75	16.1	23.0	16.40	17.8
16:1	1	3.98	4.68	0.88	1.09	1.30
	2	2.87	6.04	0.82	2.91	1.44
17:0:1	1	3.28	5.04	1.11	1.27	1.41
	2	2.55	2.87	1.07	1.27	1.41
18:0	1	12.81	8.22	11.70	12.45	12.24
	2	11.55	11.29	14.78	11.15	12.15
18:1	1	14.29	20.4	14.05	14.95	14.85
	2	14.93	17.76	15.43	15.09	17.17
18:2	1	8.48	9.65	11.05	11.61	11.57
	2	9.96	10.78	12.67	11.13	10.74
18:3	1	1.7	0.57	0.40	0.69	0.52
	2	0.88	0.8	0.11	0.19	0.26
20:0	1	--	0.22	0.57	0.45	0.63
	2	--	0.2	0.33	0.49	0.34
20:1	1	--	0.66	0.84	0.79	0.96
	2	--	1.07	0.61	0.95	0.81
20:2	1	--		0.63	0.51	0.43
	2	--		0.35	0.55	0.56
20:3	1	1.64	1.66	2.53	1.96	2.17
	2	1.16	1.31	1.50	1.57	2.22
20:4	1	15.03	5.77	15.33	13.66	11.7
	2	14.95	10.93	14.04	14.75	14.15
20:5	1	1.45	2.05	4.06	4.64	3.80
	2	1.03	4.23	1.08	3.00	2.40
22:1	1	--	0.83	2.75	2.80	3.82
	2	--	1.5	1.89	3.00	3.14
22:4	1	5.4	1.71	0.52	0.53	0.54
	2	5.65	1.27	0.83	0.50	0.46
24:0	1	3.55	0.18	0.23	0.19	0.18
	2	4.51	0.22	0.18	0.22	0.22
22:5	1	2.6	1.0	2.91	2.13	2.05
	2	2.32	1.31	1.77	2.30	2.22
22:6	1	5.42	2.46	9.40	6.99	6.45
	2	6.41	4.1	7.10	8.26	8.05
24:1	1	--	0.73	1.10	0.84	0.94
	2	--	0.63	0.94	1.00	1.02
Sum of omega 3	1	11.17	6.08	16.77	14.31	12.88
	2	10.64	10.44	10.23	13.75	12.37
Sum of PUFAs	1	50.2	25.82	46.83	42.59	39.43
	2	42.36	35.5	38.94	42.26	40.56
Sum of UFAs	1	37.18	47.03	31.83	35.6	36.68
	2	37.33	34.55	40.27	33.17	34.12

1 – Before Treatment, 2 – After Treatment

PUFAs – Polyunsaturated fatty acids

UFAs – Unsaturated fatty acids

The treatment based on a basic diet along with the inclusion of amaranth oil brought about a dose-dependant action in respect to oleic acid (18:1), the content of which increased to the maximum (by 16%) when using a 6 ml of amaranth per day. The concentration of linoleic (18:2) and linolenic acids (18:3) decreased in the course of diet therapy in patients of all the groups. Along with this, a dose-dependant action in respect to a long chain polyunsaturated fatty acid of omega family 3 – docosapentaenoic (22:5) and docosahexaenoate (22:6) was established. If the concentration of these acids in erythrocyte membranes decreased in patients suffering from coronary heart disease and hypertension who received a diet with a content of 6 ml of amaranth oil per day, then the concentration of erythrocyte membranes increased by 9% and 18% in patients of the 2^nd ^main group and by 9% and 28% in patients of the 3^rd ^main group.

The total increase in membranes polyunsaturated fatty acids was evident only in patients of the 3^rd ^main group. Simultaneously, in the course of diet therapy a maximal decrease of part unsaturated fatty acids in erythrocyte membranes.

Hence, changes in the fatty-acid composition of erythrocyte membranes under the influence of various doses of amaranth oil are evident and were only expressive when using 18 ml of oil on a daily basis. Apparently, the observed clinical effects were related not only to the change in fatty-acid composition of diet, but also the squalene content in it.

### Estimation of the antioxidant action of amaranth oil in patients with CVD

Antioxidants are important components of nutrition which prevent the formation of free radicals, inhibit them or participate in their destruction process, help to avoid oxidative stress, and provide protection from chronic diseases [52].

Amaranth grain and its oil fraction have antioxidative effect on streptozotocin-induced diebetic rats.^53 ^The authors suggest that amaranth grain and amaranth oil supplements, as an antioxidant therapy, may be beneficial for correcting hyperglycemia. However, there are not enough investigations about amaranth oil antioxidant properties specifically for humans. Below are the results of our study about amaranth oil antioxidant properties for humans.

As can be seen in Table 10, the enrichment of food ration by amaranth oil had an essential positive effect on the peroxide oxidation of lipids – antioxidant protection (POL -AP), manifesting by reduction in the products of the peroxide oxidation of lipids (diene conjugates and the malonic dialdehyde of plasma) and by growth of the ferments of antioxidant protection (glutathione reductase (GR), glutathionperoxidase(GP), superoxidedismutase (SODM), catalase(CAT).

**Table 10:** Dynamics of the indices of POL – AP system in Patients with Ischemic Heart Diseases and Hyperlipidemia (M ± m)

**Indices**		**HAD**	**Amaranth Oil **3 g (100 mg squalene per day)	**Amaranth Oil **6 g (200 mg squalene per day)	**Amaranth Oil **12 g (400 mg squalene per day)	**Amaranth Oil **18 g (600 mg squalene per day)
DC, nmole/ml	1	4.12 ± 0.34	4.98 ± 0.39	5.71 ± 0.77	5.35 ± 1.05	5.49 ± 0.49
	2	3.75 ± 0.29	4.13 ± 0.27	4.06 ± 0.42	5.07 ± 0.71	5.45 ± 0.81
MDA, nmole/ml	1	3.02 ± 0.17	4.4 ± 0.28	2.37 ± 0.34	2.688 ± 0.39	2.5 ± 0.49
	2	2.66 ± 0.08	3.38 ± 0.19	1.27 ± 0.12	2.118 ± 0.36	2.49 ± 0.49
GP, micromole/min/ml	1	20.6 ± 0.62	22.3 ± 3.77	23.4 ± 3.43	26.2 ± 1.61	24.1 ± 4.16
	2	20.4 ± 0.51	22.6 ± 3.61	23.88 ± 4.45	28.5 ± 0.99	22.4 ± 0.78
GR, micromole/min/ml	1	1.81 ± 0.14	1.77 ± 0.1	1.158 ± 0.42	0.57 ± 0.1	1.31 ± 0.18
	2	1.97 ± 0.09	1.98 ± 0.27	1.7 ± 0.98	0.8 ± 0.34	1.58 ± 0.74
SODM nominal units/ml	1	2816 ± 160	1826 ± 42.4	2112 ± 83.96	1991 ± 34.63	1998 ± 43.1
	2	3098 ± 148	1810 ± 26.3	2365 ± 77.12	2267,8 ± 70.0	2338 ± 100
CAT, kU/ml	1	183 ± 11.3	203 ± 33.3	223 ± 23.53	226 ± 17.23	178 ± 37.88
	2	226 ± 9.93	225 ± 25.8	281.8 ± 20.87	208.8 ± 4.2	244 ± 30.66

1 – Before Treatment, 2 – After Treatment

Diene Conjugates (DC), Malonic Dialdehyde (MDA), Peroxide Oxidation of Lipids – Antioxidant Protection (POL -AP), Glutathione Reductase (GR), Superoxidedismutase (SODM), catalase (CAT).

Thus, in particular, the diet therapy resulted in the reduction in the diene conjugates of plasma (DC) and of malonic dialdehyde (MDA) in the basic groups of patients respectively to 17–29% and 21–46%, accordingly. In the patients of the comparison group, the reduction in the data of indices composed 9% and 12%.

It is noted also the considerable growth of the ferments of antioxidant protection proportional to the concentration of amaranth oil in the diet. In this case, the greatest dynamics are fixed in a change in the levels of glutathione reductase (GR) and of catalase (CAT) in basic groups to 12%, 47%, 42%, 21% and 11%, 26%, 8%, 37%, and in the patients of the group of comparison to 10% and 23%, accordingly.

### The estimation of the immunomodulating action of amaranth oil in patients with ischemic heart diseases and hyperlipidemia

Humoral immunity has been suggested to play an important role in the diseases for both adults and children, but the function of neutralizing antibody responses in delaying disease progression has not been fully established [54-58].

The results of investigating the indices of humoral immunity are given in Table 11.

**Table 11:** Dynamics of the Indices of Humoral Immunity in Patients with Ischemic Heart Diseases and hyperlipidemia under the effect of the basic therapy with the application amaranth oil (M ± m)

**Indices**		**HAD**	**Amaranth Oil **3 g (100 mg squalene per day)	**Amaranth Oil **6 g (200 mg squalene per day)	**Amaranth Oil **12 g (400 mg squalene per day)	**Amaranth Oil **18 g (600 mg squalene per day)
IgM, mg/ml	1	0.79 ± 0.07	0.81 ± 0.07	2.25 ± 0.96	1.82 ± 0.78	1.0 ± 0.33
	2	0.73 ± 0.03	0.87 ± 0.06	2.43 ± 1.04	1.98 ± 0.78	1.2 ± 0.21
IgG, mg/ml	1	4.32 ± 0.37	4.33 ± 0.97	8.25 ± 0.45	5.56 ± 1.86	11.28 ± 1.76
	2	4.45 ± 0.7	5.63 ± 0.1	10.23 ± 3.89	7.33 ± 2.66	15.27 ± 0.13
IL-4, pg/ml	1	13.7 ± 2.8	8.91 ± 1.9	15.0 ± 3.3	7.67 ± 2.19	12.75 ± 4.66
	2	15.64 ± 2.2	11.0 ± 3.2	17.78 ± 5.79	12 ± 3.22	16.5 ± 2.51
IL-1β, pg/ml	1	173.9 ± 20.97	186.4 ± 25.3	8.0 ± 1.41	4.0 ± 1.22	4.32 ± 1.16
	2	150.7 ± 19.52	112.6 ± 21.32	4.62 ± 1.53	2.84 ± 1.02	2.38 ± 0.06

1 – Before Treatment, 2 – After Treatment

According to the immunogram, the levels of IgM and IgG content in the blood serum of patients before treatment and after treatment was within the norm (0,5–2,0 g/l for IgM; 5,3–16,5 g/l for IgG).

It is known that Il-1β appears cytokin of the wide spectrum of action, produced predominantly by macrophages. It causes the starting reactions of immunity and plays a key role in the development of inflammation. Il-1β also plays one of the central roles in the inflammatory reaction, in response to the bacterial infection and the tissue damages.

Raising the level of Il-1β is observed with different inflammatory and autoimmune diseases, including cardiovascular pathology. Statistically reliable reduction of its content in the blood serum in patients, who had an amaranth oil intake, contained respectively 100, 200, 400 and 600 mg of squalene (to 39%, 42%, 28% and 45%) and an improvement in the clinical state of patients. Furthermore, patients who had a standard diet intake had a 14% reduction.

The level of free fraction Il-4 in the blood serum of patients who consumed amaranth oil exceeded initially to 26%. After the course of treatment, there was an increase in the content of cytokin in the blood serum of patients who consumed amaranth oil, contained 100, 200, 400 and 600 mg of squalene, accordingly to 24%, 19%, 57% and 29% (r<0,05). Furthermore, patients who had a standard diet intake had a 14% reduction. As primary targets, Il-4 serves B-lymphocytes for which it is the strongest growth factor. In other words, there was an improvement in the humoral immunity of patients. It was then established that amaranth oil possesses high biological activity.

This is true because amaranth oil contains 77% of polyunsaturated fatty acids, from which 50% comprises the linoleic acid. Furthermore, amaranth oil is characterized by the high content of such biologically active compounds, such as: squalene (up to 8%), tocopherols (to 2%), phospholipids (to 10%), and phytosterols (to 2%). A good germicidal action is established, and also the high regeneration and antitumorigenic properties of amaranth oil. Amaranth oil increases the antioxidant and immunomodulating property of HAD.

In conclusion, the results of the present study shows that amaranth oil can reduce the amount of cholesterol in blood serum, and it can be recommended as a functional food product for the prevention and treatment of cardiovascular diseases. Diet with amaranth oil may help reduce blood pressure and could serve as an effective alternative to drug therapy in people with hypertension. This investigation also showed that a combination of amaranth oil with a hyposodium antiatherogenic diet is more effective to reduce the amount of blood cholesterol than just the hyposodium antiatherogenic diet. The next step of our investigation is to study mechanism of cholesterol lowering properties of amaranth oil. Squalene as well as phytosterolls can be involved in that mechanism. As you can see on the Table 4 amaranth oil contains great amount of phytosterols, which are chemical homologs of cholesterol. Phytosterols interfere with the micellar solubilization of cholesterol in the intestine and reduce the efficiency of cholesterol absorption [59].

## Conclusion

1. The inclusion of amaranth oil in the diet has a beneficial action upon the clinical presentation of Coronary Heart Disease and Hypertension. Its beneficial action is seen best when used at a dose of 18 ml per day.

2. Amaranth oil decreases the amount of total cholesterol, triglycerides, LDL and VLDL significantly. The concentration-dependent cholesterol lowering effect of characterizing amaranth oil has been proven.

3. The inclusion of amaranth oil in the diet contributes to an increase in the concentration of polyunsaturated fatty acids, particularly, long-chain acid of omega 3 families in patients suffering from hypertension and coronary heart disease.

4. Our studies indicate that Amaranth oil can be considered as an effective natural antioxidant supplement capable of protecting cellular membranes against oxidative damage.

## Abbreviations

Hyposodium Antiatherogenic Diet (HAD), Atherogenic Coefficient value (AC), Diene Conjugates (DC), Malonic Dialdehyde (MDA), Peroxide Oxidation of Lipids – Antioxidant Protection (POL -AP), Glutathione Reductase (GR), Superoxidedismutase(SODM), Catalase(CAT), Eicosapentaenoic Acid (EPA), Docosahexaenoic Acid (DHA), PUFAs – Polyunsaturated Fatty Acids, UFAs – Unsaturated Fatty Acids
